# Immunopeptidomic Data Integration to Artificial Neural Networks Enhances Protein-Drug Immunogenicity Prediction

**DOI:** 10.3389/fimmu.2020.01304

**Published:** 2020-06-23

**Authors:** Carolina Barra, Chloe Ackaert, Birkir Reynisson, Jana Schockaert, Leon Eyrich Jessen, Mark Watson, Anne Jang, Simon Comtois-Marotte, Jean-Philippe Goulet, Sofie Pattijn, Eustache Paramithiotis, Morten Nielsen

**Affiliations:** ^1^Immunoinformatics and Machine Learning, DTU Health Technology, Danish Technical University, Lyngby, Denmark; ^2^ImmunXperts, Gosselies, Belgium; ^3^Caprion Biosciences, Montreal, QC, Canada; ^4^IIBIO-UNSAM, Universidad Nacional de San Martin, Buenos Aires, Argentina

**Keywords:** MHC-II prediction, machine-learning, protein-drug immunogenicity, artificial neural-networks, immunopeptidomics, bioinformatics

## Abstract

Recombinant DNA technology has, in the last decades, contributed to a vast expansion of the use of protein drugs as pharmaceutical agents. However, such biological drugs can lead to the formation of anti-drug antibodies (ADAs) that may result in adverse effects, including allergic reactions and compromised therapeutic efficacy. Production of ADAs is most often associated with activation of CD4 T cell responses resulting from proteolysis of the biotherapeutic and loading of drug-specific peptides into major histocompatibility complex (MHC) class II on professional antigen-presenting cells. Recently, readouts from MHC-associated peptide proteomics (MAPPs) assays have been shown to correlate with the presence of CD4 T cell epitopes. However, the limited sensitivity of MAPPs challenges its use as an immunogenicity biomarker. In this work, MAPPs data was used to construct an artificial neural network (ANN) model for MHC class II antigen presentation. Using Infliximab and Rituximab as showcase stories, the model demonstrated an unprecedented performance for predicting MAPPs and CD4 T cell epitopes in the context of protein-drug immunogenicity, complementing results from MAPPs assays and outperforming conventional prediction models trained on binding affinity data.

## Introduction

The advent of recombinant DNA technology in the last decades has boosted the use of protein drugs as pharmaceutical agents. However, a major potential problem of these—compared to lower molecular weight pharmaceutical counterparts—is adverse effects associated with protein immunogenicity. Immunogenicity is generated because the drug is recognized as non-self, involving an unwanted activation of CD4 T cells, and the formation of anti-drug antibodies (ADAs), potentially producing a hypersensitivity reaction in treated patients.

Protein drug activation of CD4 T cells depends on the internalization of the drug into endosomal compartments in antigen-presenting cells (APCs), where proteolytic enzymes digest the protein into smaller peptides (1). According to specific rules, a small proportion of those peptides are loaded into major histocompatibility complex class II (MHC-II) molecules. Then, stable peptide-MHC-II complexes are exported to APCs' surface for presentation to CD4 T cells, which can initiate, maintain, and regulate immune responses, including the production of ADAs (2). As a consequence, finely characterizing the rules of MHC-II binding and antigen presentation is of high interest to promote a general understanding of T cell immunogenicity and for the development of biotherapeutics.

Each MHC-II complex has distinct peptide-binding preferences predominantly determined by residues in the MHC binding groove. The MHC binding groove interacts with a stretch of 9 amino acids termed the peptide core. For every MHC-II molecule, a few pockets accommodate specific positions of the peptide core with a narrow or broader specificity for different residues (3). These pockets and pocket specificities are dependent on the class II molecule of study. MHC-II in humans comprises three major gene pairs called HLA-DR, -DP, and -DQ, all having an α- and a β-chain. The MHC presentation of peptides is fundamentally determined by the amino acid sequence of the peptide and the MHC-II alleles expressed by the host. However, other factors, such as protein internalization or peptidase cleavage sites, influences which peptides are presented.

Historically, peptide-MHC binding affinity (BA) measurements have been used to characterize MHC binding preferences (4), and collections of BA data have been used to develop methods such as NetMHCII and NetMHCIIpan (5–8) with the ability to predict peptide binding to different MHC class II molecules. However, the predictive power of these methods for CD4 T cell epitopes remains limited. Recently, the introduction of ligandome data as obtained by mass spectrometry (MS) immunopeptidome assays (9) has improved MHC predictors' performance substantially (10–14).

Analyzing MS-data has allowed us to learn the rules of MHC-II peptide presentation beyond peptide-MHC binding, including peptide cleavage specificities. The incorporation of such data to MHC-II models has demonstrated to improve state-of-the-art prediction for “natural binders” (14). Currently, MHC-associated peptide proteomics (MAPPs) are used to assess the immunogenicity of protein drugs (15, 16). However, several factors entangle assay performance and interpretation. First, most of the peptides detected by MAPPs are of self-origin, and only a small fraction of the peptides come from the protein drug of interest. Thus, to increase the sensitivity toward the given protein of interest, the amount of sample required is very high, which can lead to aggregation of the protein drug *in vitro*, changing the immune response (17, 18). Second, although MS sensitivity has increased over the past years, still the comprehensive analysis of the peptide ligandome is highly challenging, making it necessary to perform several technical replicates to obtain the maximum amount of peptides identified (19–21). In addition, variations in MHC alleles dictate which peptides will be presented in a given MAPPs context, making necessary the study of several donors with different alleles, representing the population of interest, to accurately assess immunogenicity. Because of those reasons, learning the specific rules of MHC-II presentation in the form of an *in-silico* predictor would constitute a definite step forward in the development of means to assess the immunogenicity of protein drugs effectively.

Recently, several publications have integrated MS data into MHC-II predictors applying different machine learning approaches (22–26). As regular cells can express up to 12 different HLA alleles including the HLA-DR, -DQ, and -DP genes, a large challenge of this integration lies in how to assign ligands to their HLA restriction element.

To tackle this question, different strategies have been proposed. Abelin et al. (24) used an experimental approach transfecting cells with modified HLA molecules able to be independently purified with a biotin-avidin system to perform “single allele” (SA) mass spectrometry. The peptides derived from each are then used to train allele-specific prediction models. The main disadvantage of this method is the limited set of predictable MHC-II alleles. Chen et al. (26) used a multimodal recurrent neural network to predict MHC class-II ligands, integrating binding affinity, mass-spectrometry data, and RNAseq expression levels. A recurrent neural network was trained on binding affinity data only to resolve the ligand HLA restriction. This method however did not show improved performance over netMHCIIpan, suggesting that Deep neural networks not necessarily outperform shallow neural networks in MHC-II prediction. This method was further suggested optimal for neoepitope discovery, where protein expression is relevant, a factor that is not applicable for prediction of protein drug immunogenicity. Finally, MixMHC2pred from Racle et al. (25) used a probabilistic framework to deconvolute MHC-II peptidomics to the specific allele, and after used a method based on scoring matrices for prediction, using a small set of relevant HLA-DR alleles. None of these recent methods, however, are pan-specific nor were conceived or previously used to predict protein drug immunogenicity.

We have recently developed a neural network framework, NNAlign_MA, that is able to deconvolute mass spectrometry data and at the same time train a predictor to learn the binding preferences of individual MHC molecules (22, 23, 27). In this work, we have trained an immunogenicity predictor based on this NNAlign_MA framework integrating ligand information obtained from in-house Infliximab MAPPs assays, and binding affinity measurements to build a prediction model for MHC-II antigen presentation. Using this model as a proxy for immunogenicity prediction, we showcase its performance on Infliximab and Rituximab, two well-known protein drug antibodies used to treat inflammatory diseases and known to generate an unwanted immune response (10–60% according to the analyzed disease, and how and when immunogenicity is screened) (28–30).

## Materials and Methods

### Samples

#### Donors and Alleles

Peripheral blood mononuclear cells (PBMCs) were isolated from leukapheresis donated by seven healthy volunteers (ethical protocol IXP-004 Belgium; Reg. Nr. B707201629385). Monocytes were isolated by positive magnetic separation and cultured for 5 days in DC medium supplemented with interleukin 4 (IL-4) and granulocyte-macrophage colony-stimulating factor (GM-CSF). Immature dendritic cells (iDCs) were pulsed with Infliximab at 50 μg/ml and further matured with Lipopolysaccharide (LPS) for ~20 h. Mature DCs (mDC) were collected, counted and washed with Dulbeco's Phosphate Buffered Saline (DPBS), and stored at −80°C as dry pellets without supernatant.

Allele genotypes of the donors were defined using Sequence-Based Typing (SBT) and are detailed in Supplementary Table 1.

#### Proteins and Peptides

Infliximab (Inflectra) was acquired from Hospira®. Peptides screened for T cell activation were purchased from Mimotopes and are listed in Supplementary Table 2.

### MHC-Associated Peptide Proteomics (MAPPs) Assay

#### Cell Lysis

Dendritic cell pellets (1–6 million cells) were lysed in non-ionic detergents (4% CHAPS and 4% Triton X-100) in the presence of protease inhibitors (EDTA-free, Roche) and 590 units of nuclease (US Biologicals) for 45 min at 4°C with rotation. The cell lysate was clarified by centrifugation at 112,000 g for 30 min at 4°C.

Immuno-isolation of MHC II complexes. An isotype IgG (Southern Biotech) and the pan anti-MHC II class monoclonal antibody (L243) (BioXCell) were each coupled to individual HiTrap NHS-activated HP columns (GE Healthcare). The two columns were connected in series with the Isotype IgG column first for the immuno-isolation process. The cleared lysate was loaded on the immuno-isolation columns. The Isotype IgG column was removed, and the MHC II complexes were washed with a buffer and then eluted from the L243 column with 10% acetic acid. The MHC II peptides were desalted by solid-phase extraction using an MCX plate (Waters) into LoBind 96 well plates (Eppendorf) and then transferred to MS plates (Abgene), and vacuum evaporated.

### Mass Spectrometry Analysis

Peptide samples were re-solubilized with 10 μL solubilization buffer [96/4 (v/v) water/acetonitrile (CAN) + 0.2% formic acid + 25 mM TCEP (Tris(2-carboxyethyl)phosphine)]. 7 μL were injected on a Waters nanoACQUITY UPLC system, and peptide separation was achieved with a Symmetry C18 trap column (100 Å, 180 μm x 20 mm, 5 μm particle size) and a BEHC18 column (300 Å, 150 μm x 100 mm, 1.7 μm particle size) coupled to a Q-Exactive Plus mass spectrometer (Thermo). Peptides were eluted with an ascending acetonitrile gradient over 105 min. MS spectra were acquired from 400 to 1,800 Da. The MS method consisted of a full MS scan followed by a dd-MS2 scan of the top 12 ions. The full MS scan was achieved with a resolution of 70,000 with an AGC value of 3 × 10^6^ and a maximum IT level of 30 ms. The dd-MS2 scan was performed at a resolution of 17,500 with an AGC value of 5 × 10^4^ and a maximum IT level of 60 ms. Blank runs of resolubilization-buffer were injected between each sample.

### MS Data Processing and Peptide Identification

A single custom database of protein sequences relevant to the experiment was created to include the Human proteome (Swissprot), common general and Caprion-specific laboratory contaminants, and Infliximab (Inflectra) sequence.

Peak alignment and extraction of intensity values of peptide ions and corresponding MS/MS spectra were performed using Rosetta Elucidator™ (Rosetta Biosoftware, version 3.3). MS/MS spectra were then exported for peptide identification in PEAKS Studio (Bioinformatics Solutions, version 7.5). Search parameters included the custom database described above, non-tryptic, oxidation of methionine and deamidation of asparagine as variable modifications, and error tolerance of 15 ppm for precursor mass and 0.025 Da for fragment ions. Data were filtered using a 2% FDR at the peptide level for database search results.

The mass spectrometry proteomics data have been deposited to the ProteomeXchange Consortium via the PRIDE partner repository (http://www.ebi.ac.uk/pride) with the dataset identifier PXD018303.

### Neural Network Architecture and Datasets

#### Training Datasets

The NNAlign_MAC model was trained combining multi-allele (MA), and single-allele (SA) data including binding affinity (BA) peptide measurements and mass spectrometry (MS) data.

MA datasets included only self-protein MS eluted ligands obtained from in-house MAPPs assays. The alleles expressed by each donor are detailed in Supplementary Table 1. Infliximab and Rituximab are chimeric antibodies that bear the constant region from a human antibody. Therefore, it is expected that some naturally presented peptides share similarities to the protein-drug antibodies. To avoid a bias in the predictor when evaluating the protein-drug antibodies, we have excluded all peptides sharing a common motif of 9 amino acids (defined by the length of an MHC-II binding core) to both Infliximab and Rituximab proteins from the training dataset. This resulted in the removal of 262 peptide sequences from the mass spectrometry datasets. Additionally, the data were filtered to only include peptides with lengths 13 to 21 in the training datasets.

SA data included peptides derived from BA measurements or MS assays where cells were specifically-homozygous selected or were artificially and genetically engineered to only express a single HLA-DR allele. SA data was collected from previous NetMHCIIpan publications (5, 23), and updated with IEDB to date 01/28/2019.

Mass spectrometry data consists only of “positive” presented peptides. Therefore, a set of negative peptides was added to train artificial neural networks, randomly sampling different length peptides from human proteins. For each MA donor-dataset or SA allele-dataset, a set of random negatives were included following a flat distribution of lengths 13–21, taking 5 times the number of peptides of the most abundant peptide length on the positive dataset. The flat distribution of the negatives helps the neural network to learn the natural length preference of the data, while the selection of 5 times the most abundant length will generate a ratio of ~1:10 positive to negatives, which we have previously benchmarked and found optimal (14). Although this approach will introduce some noise to the model, as it is possible that by chance some random peptides will bind to the specific MHC allele, this probability is very low and at the most will diminish the model performance.

### Five-Fold Partitioning

All the data combined (SA and MA) were clustered into 5 partitions using a Hobohm algorithm with a common motif of 9 amino acids to perform cross-validation as previously described (27). The artificial neural network architecture consists of an ensemble of 150 independent networks varying; the seeds for weight initialization (10), a different number of hidden neurons in the hidden layer (20, 40, 60), and the 5 different partitions used for cross-validation. An average of the ensembles is used for the final predictions.

### NNAlign_MAC Architecture

NNAlign_MAC algorithm integrates the basis of NNAlign_MA (22), an extension of NNAlign (27, 31), with peptide context information (PCI) (14, 23).

In short, NNAlign_MA (22) is a neural network framework capable of taking a mixed training dataset composed of SA data (peptides experimentally tested on a single MHC molecule) and MA data (peptides experimentally tested in cell lines expressing multiple MHC alleles), to fully deconvolute the specific MHC restriction of all MA peptides, while learning the binding specificity for all the MHCs alleles. The algorithm is trained in two steps. In a first step or pre-training (set-up here to 20 iterations), the neural networks are trained with SA data. After these initial iterations, the model manages to learn the first pattern for all MHC class II alleles. This is possible due to the pan-specific algorithm used here (that introduces relevant MHC amino acid positions known to participate in the interaction with the peptide in the binding groove (8). Based on this initial learning, the algorithm annotates the MA data according to the learnt binding rules. In a second step, those newly tagged MA peptides, now converted into SA with a specific MHC allele association, are included in a new training cycle of the network. As more data is included, the binding core for each MHC-II allele is revised. After each new training cycle, all the MA peptides are re-annotated to SA data again. This process is iterated up to 400 training cycles, thus refining the process until convergence (22).

The input neurons of this model were fed with: the peptide sequence (tagged from different experimental sources, BA or MS); a binding affinity measurement in the case of BA, or a binary classification (1-0) for those peptides derived from MS; the allele information (either single or with all alleles expressed by the donor-dataset for MA); and the MHC pseudo-sequence (specific positions of the MHC protein sequence involved in the MHC-peptide recognition). This training resulted in a pan-specific model with the power to infer binding specificities also for the HLA-DR molecules not included in the training datasets. Additionally, a separate set of input neurons encoded peptide length and peptide context information (PCI) as described elsewhere (14). PCI included 3 amino acids from both C and N peptide termini (previously named peptide flanking regions) and 3 amino acids both from upstream and downstream of the MS peptide protein sequence.

### Cross-Validation Performance

After training both models (with and without PCI), the test sets were predicted, and an AUC 0.1 calculated for each MA-donor-dataset and reported in Supplementary Table 3.

NetMHCIIpan version 3.2 (5) prediction algorithm was employed in this work as a benchmark comparison to the NNAlign_MAC model. As it was not possible to re-train NetMHCIIpan with the same partitions used for NNAlign_MAC to report AUC 0.1, the following scheme was used. Each peptide in the NNAlign_MAC test set was predicted for all the alleles expressed by the given donor with NetMHCIIpan, and the lowest %rank score from all alleles was assigned to each peptide to perform an AUC 0.1 per donor (Supplementary Table 3).

AUC is a common performance measure for predictive models, which takes into account the relationship between true positive rates (TPR) and false positive rates (FPR) for different prediction thresholds. AUC 0.1 (area under the ROC curve integrated up to a false positive rate of 10%) is similar to AUC but focuses on the high specificity range of the ROC curve.

### Logos

Sequence logos for binding motifs and context information were constructed applying the Seg2Logo (32) tool using Kulback-Leibler logos and excluding sequence weighting. Amino acids were grouped by negatively charged (red), positively charged (blue), polar (green), or hydrophobic (black).

### Infliximab and Rituximab Performance Evaluation

#### MAPPs Profiles

Infliximab in-house MAPPs were gathered together removing peptide duplicates from the same donor and imposing a filter of a minimum of 12 amino acids to be an MHC-II binder, to build a MAPPs cohort. After filtering, 73 peptides were mapped to Infliximab protein sequences, stacking them, and counting the number of peptides covering each position. The profiles were normalized to have a maximum value of 1.

Additional Infliximab and Rituximab MAPPs peptides were collected from Hamze et al. (15). Filtering and profiles were generated in the same way as for the in-house MAPPs.

#### NNAlign_MAC Evaluation

For each HLA molecule present in the MAPPs cohort, 1 × 10^5^ random peptides—with a flat length distribution of 13–21—were predicted using NNAlign_MAC, and the N-percentile score for each estimated. For each N, a score threshold per allele was defined to select HLA binders from the protein-drug of interest to be included in the prediction profile. Subsequently, all the peptides were stacked in the protein-drug sequence and the number of peptides overlapping each sequence position was counted. After that, a so-called “allele promiscuity” calculation was applied, capping the count per allele to a maximum of 1 per position. For example, a protein sequence position, with 10 peptides mapped to it from 3 different alleles, will have promiscuity of 3. After max normalization, these values refer to the “Promiscuity score” in all the profile plots in the manuscript.

From the HLA binding profiles made for Infliximab in Supplementary Figure 1, and precision and recall curves for different N values (Supplementary Figure 4A), 1% Rank (*N* = 1) was found to be optimal.

### MixMHC2pred Evaluation

MixMHC2pred version 1.2 method was downloaded from the GitHub repository to run locally for all overlapping 13–21mers from Infliximab protein-drug and all the alleles present in the MAPPs cohort and covered by the method (HLA-DRB1^*^04:03, HLA-DRB1^*^1302, HLA-DRB1^*^15:02, HLA-DRB3^*^03:01, HLA-DRB5^*^01:02 were excluded). The output column for regular %Rank was selected. An HLA binding profile was constructed for MixMHC2pred (Supplementary Figure 2), and 0.5%Rank was selected to compare with NNAlign_MAC. After the peptides' selection, the profiles and the Promiscuity Score were generated in the same manner as for NNAlign_MAC.

### NetMHCIIpan Evaluation

To calculate NetMHCIIpan infliximab binding profiles, binding profiles were constructed for % Ranks values of 1, 2, 5, and 10 (Supplementary Figure 3). The performance of NetMHCIIpan was consistently found to be very low and close to random for all % Rank thresholds for both protein chains with only one example (% Rank of 2, LC) demonstrating a positive correlation to the MAPPs profile. Given this, a value of %Rank of 2 was selected for this method. After the peptides' selection, the profiles and the Promiscuity Score was generated in the same way as for NNAlign_MAC.

### Performance Measures

Two types of correlations were used to compare predictions from NNAlign_MAC, MixMHC2pred, and NetMHCIIpan to experimental MAPPs profiles. First, the Spearman correlation coefficient (SCC) was used to correlate the profiles' predictions to MAPPs per position in the protein sequence. Additionally, scatter plots were made to confirm the correlation after losing positional information that could bias our interpretation. The scatter plot correlation was measured both using SCC and Pearson's coefficient correlation (PCC).

Additional measures, PPV and AUC0.1, were used to compare performance across the methods in Supplementary Figure 4B. To allow for minor inconsistencies between the predicted and actual positive peptides, we here adapted a relaxed definition of positives. This was done by assigning all predicted binders (as defined by the selected % Rank threshold) with a binding core that overlapped any of the “original” MAPPs peptides as positive. This set of peptides is termed the “expanded-core” MAPPs peptides. Next, this set of expanded-core peptides is used to calculate AUC0.1 (area under the receiver operator curve integrated up to a false positive rate of 10%), and PPV values using the lowest % Rank score predictions over all the alleles expressed by the donor as prediction values for each of the peptides. PPV was calculated as the number of true positive predictions from the number of “expanded-core” MAPPs in the top N predictions, divided by N, where N is the number of positives in the “expanded-core” MAPPs dataset per donor. Precision and recall curves were likewise calculated using the “core” scheme for each of the different % Rank (Supplementary Figure 4A).

Bootstrap resampling was used to calculate p-values of the SCC correlations comparison among methods, or %Rank values. 10 thousand sampling iterations with allowed repetitions were picked at random for each comparison. The p-value was obtained by #losses/iterations, where losses reflect the number of times the SCC was higher for the challenging method over the other.

### Evaluation of the CD4-T Cell Response

PBMCs from 6 out of 7 donors were seeded at 2 × 10^6^ cells/well and stimulated with the different test and control peptides (Supplementary Table 2). For one donor, the number of cells was not sufficient to perform this assay. The next day, IL-7 was added. On day 4, part of the medium was changed and IL-2 and IL-7 were added. On day 7, cells were harvested, and rested overnight at 37°C. The next day, cells were counted and seeded in IFN-y FluoroSpot plates (Mabtech). Cells were re-stimulated with peptide or left unstimulated overnight, in duplicates. On day 9, FluoroSpot plates were developed, according to the manufacturer's instructions. Data, spot forming units (SFU), were acquired with a Mabtech IRISTM FluoroSpot/EliSpotReader. Raw data (SFU) were transferred to SFU per million, which were then transferred to ΔSFU per million. ΔSFU per million = Average SFU peptide condition/per million-Average medium condition/per million.

We defined a positive response when the two independent peptide measurements were 4 standard deviations higher to the average signal for the control. Raw data, averages and statistical calculations are included in Supplementary Table 4.

An additional dataset of T cell responses for Infliximab (30 epitopes from 21 donors) and Rituximab (14 epitopes from 16 donors) was collected from Hamze et al. (15).

## Results

Here, we aimed to develop a predictor for MHC class II antigen presentation and assessed its performance for prediction of protein-drug specific MAPPs readouts and T cell epitopes.

### NNAlign_MAC Is Able to Predict Infliximab-Associated MAPPs in a Cohort-Based Approach

**Figure 1:**
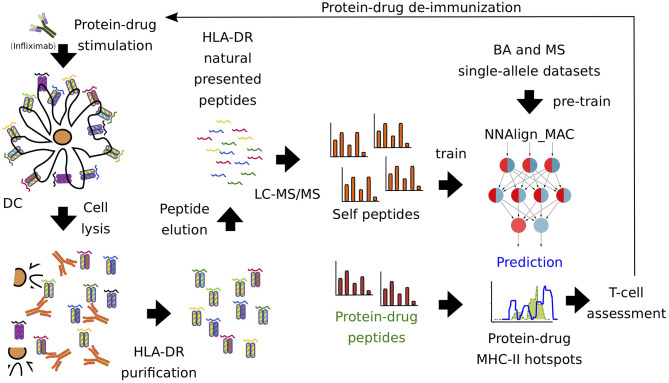
Pipeline of protein-drug (Infliximab) immunopeptidome profiling. Infliximab-pulsed DCs were lysed and HLA-DR-peptide complexes were purified with a pan-specific antibody (L243). Next, LC-MS/MS was performed, identifying 15,240 unique ligands. MAPPs self-proteins were used to train the artificial neural network model, NNAlign_MAC. Infliximab MAPPs peptides were pooled from different donors and used to compare to the predicted MHC-II hot-spots regions. Finally, T cell experiments were used to validate regions and select protein-drugs residues prone to introduce modifications in order to avoid immunogenicity.

First, we sought to profile the MHC class II immunopeptidome of Infliximab (as a biotherapeutic prototype) to predict the immune response associated with it. For that purpose, we pulsed with Infliximab 7-donor monocyte-derived dendritic cells, expressing the most common world population HLA-DR alleles (Supplementary Table 1). Next, LC-MS/MS was performed, identifying 15,240 unique ligands. After removing ligands with a common motif to Infliximab and Rituximab protein sequences (see Materials and Methods), the remaining dataset was combined with single-allele BA and MS data collected from IEDB, to construct a dataset for training a model for HLA-DR antigen presentation prediction (Figure 1).

This training was performed using the NNAlign_MA machine learning framework allowing for accurate deconvolution of HLA-DR binding specificities and proper assignment of each MS ligand to its likely HLA-DR restricting molecule (22). Earlier work has shown this algorithm to be able to accurately perform this task, and at the same time to learn the rules for the MHC-II motifs present in the samples (22, 23). The algorithm used here was extended to include “peptide context information” (PCI) from the peptide flanking regions (PFRs) on both peptide termini, and from the protein sequence upstream and downstream the MS peptide sequence. The introduction of PCI was previously shown to significantly reinforce the learning of the rules of “natural processing” in the model (14). Evaluating the predictive power of models trained with and without PCI inclusion, confirmed this earlier observation (Supplementary Table 3). This benchmark also confirmed a consistent and very pronounced gain in prediction performance of the NNAlign_MA method compared to the state-of-the-art method, trained with binding affinity measurements, NetMHCIIpan, for prediction of MHC eluted ligand data (Supplementary Table 3). We termed the NNAlign_MA model including PCI, NNAlign_MAC.

**Figure 2:**
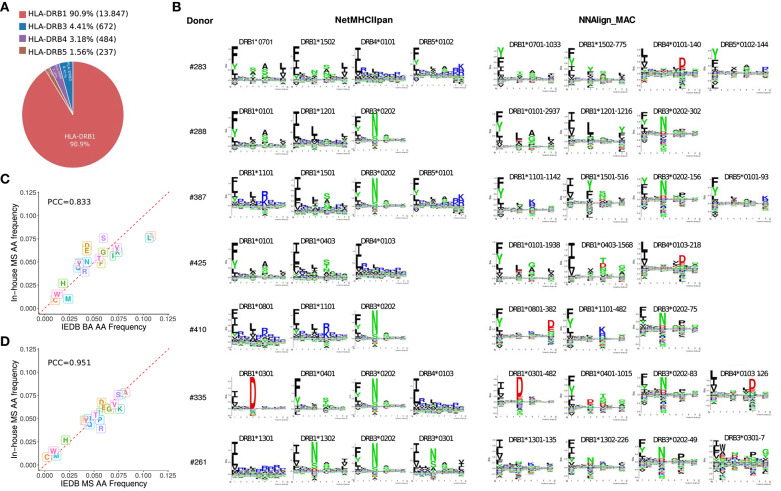
HLA-DR peptide distribution, binding motifs and amino acid frequencies. **(A)** MAPPs peptide frequency from all donors combined associated to each HLA-DR gene (HLA-DRB1, DRB3, DRB4, and DRB5) after NNAlign_MAC deconvolution. Percentages (and absolute numbers) are shown for the peptides assigned to each allelic variant. **(B)** Motif deconvolution obtained by NNAlign_MAC per donor. NNAlign_MAC allele logos were built with all peptides from each MS data set assigned for that particular allele. The number after the allele name reflects the number of peptides found in that dataset for the given allele (Example: DRB3^*^03:01-7 peptides). NetMHCIIpan motifs were built from top 1% scoring prediction of 100,000 random peptides evaluated using the list of alleles expressed in each donor sample. Motif logos were build using Seq2Logo with default settings. **(C,D)** Amino acid frequency comparison of in-house MAPPs and peptides from binding affinity (BA) assays **(C)** and mass spectrometry (MS) eluted ligands **(D)** collected from IEDB. For each comparison, 500 peptides per allele were selected at random per each allele (DRB1^*^01:01, DRB1^*^04:01, DRB1^*^07:01, DRB1^*^11:01, and DRB1^*^15:01) and pooled together before the amino acid frequency was calculated.

After deconvolution, each MS ligand was annotated to a specific allele expressed in the sample assessed. As expected, HLA-DRB1 due to the higher expression of those genes compared to HLA-DRB3, −4 and −5 (33), was assigned the highest proportion of ligands (~90%, Figure 2A). All the motifs obtained by NNAlign_MAC share a remarkable overall correspondence across cell samples expressing the same alleles, and to a lesser degree, also with the NetMHCIIpan motifs (Figure 2B). The HLA-DRB4^*^01:03 allele was shared by two donors, and the motifs obtained by NNAlign_MAC in these two, shared highly similar amino acid preferences (PCC = 0.924). Additionally, for some alleles, such as HLA-DRB1^*^08:01 and HLA-DRB4^*^01:03, the motifs from NetMHCIIpan and NNAlign_MAC, were however discordant (Figure 2B). Comparing the amino acid composition of the in-house MS data to that of MS data obtained from IEDB revealed a high consistency between the two MS datasets (PCC = 0.95) and a lower consistency to the BA data (PCC = 0.83), supporting the quality of in-house MS data, and suggesting that MS data may contain complementary information to BA data (Figures 2C,D).

**Figure 3:**
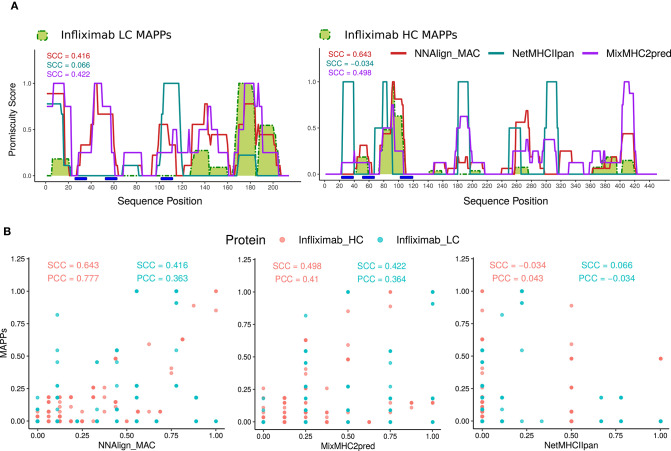
NNAlign_MAC improves Infliximab MAPPs predictions. **(A)** Infliximab profile predictions were generated with NNAlign_MAC (red), MixMHC2pred (purple), and NetMHCIIpan (blue), and benchmarked against Infliximab experimental MAPPs (green). Promiscuity profiles were generated for each method, selecting the protein-drug predicted peptides below a defined %Rank threshold, and stacking the peptides over the protein sequence (see section Materials and Methods). The correlation of the different profiles to MAPPs data (Spearman correlation coefficient, SCC) is shown in matching colors for each prediction method. Different %Rank values were selected for each method according to its best predictive power (NNAlign_MAC = 1, MixMHCIIpred = 0.5, and NetMHCIIpan = 2) (Supplementary Figures 1–3). Complementarity determining regions (CDRs), were calculated with the DomainGapAlign tool of IMGT.org (CDR1-IMGT:27-38; CDR2-IMGT: 56-65; CDR3-IMGT:105-117), both for infliximab heavy and light chain variable domains (blue rectangles). **(B)** Scatter plots of the predicted profiles in **(A)** for NNAlign_MAC, MixMHC2pred, and NetMHCIIpan vs. MAPPs. Both SCC and PCC are shown for Infliximab heavy (Infliximab_HC, red) and light chain (Infliximab_LC, blue). The discrete patterns in the x-axis of the plots are explained by the maximum number of alleles predicted for each method (MixMHC2pred is only available for a limited set of alleles).

Next, all MAPPs peptides unique to infliximab (73 peptides from 7 donors) were mapped to the heavy and light chains of the protein-drug and the count of peptides overlapping each amino acid position in the protein sequence was used to build a MAPPs profile (normalized to have a maximum value of 1) (Figure 3A). Later, infliximab sequences were (*in-silico*) digested into overlapping 13–21mer peptides, and the likelihood for MHC presentation predicted for each peptide using NNAlign_MAC, MixMHC2pred, or NetMHCIIpan for all the HLA-DR alleles present in the donor cohort (Supplementary Table 1). To define a threshold defining positive predicted peptides from each of the models, HLA binding-profile analyses were performed for different % Rank thresholds for each of the models (Supplementary Figures 1–3). Based on these analyses, a Rank threshold of 1% was selected for NNAlign_MAC, a 0.5% MixMHC2pred, and a value of 2% for NetMHCIIpan (detailed in Materials and Methods section, Supplementary Figures 1–3, 4A).

For each predicted HLA molecule, all the peptides with predicted values below the selected % Rank threshold were mapped to the Infliximab heavy and light protein sequences. Next, each position in the protein sequence was assigned a value of 1 if it was covered by one peptide or more and zero otherwise. Finally, these allele-specific binary peptide-maps were stacked constructing a “promiscuity profile” reflecting how many different alleles presented peptides overlapping a given protein position (detailed in Methods) (Figures 3A,B). This mapping was performed for each of the three prediction methods (Supplementary Figures 2, 3, respectively). Comparing the predicted profiles and experimental MAPPs demonstrated an improved power of NNAlign_MAC (SCC = 0.416 and SCC = 0.643) compared to NetMHCIIpan (SCC = 0.066 *p*-value <10-4 and SCC = −0.034 *p*-value = 0.0004) for predicting infliximab MAPPs data. And an improved power compared to MixMHC2pred for the heavy chain (SCC = 0.498 *p*-value <10-4), and a comparable power compared to the light chain (SCC = 0.422 *p*-value = 0.802). All regions in Infliximab covered by MAPPs were identified by NNAlign_MAC and MixMHC2pred. In contrast, NetMHCIIpan failed to predict several of these regions (one prominent example being the region spanning positions 40–60 in the heavy chain).

**Figure 4:**
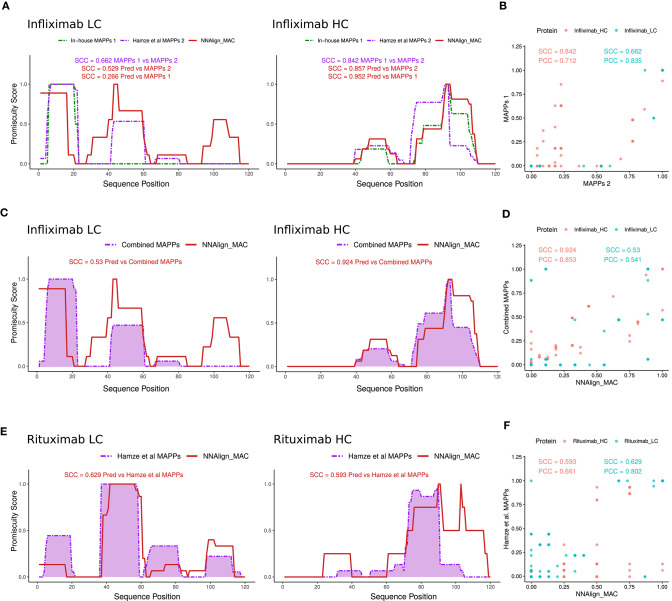
NNAlign_MAC Infliximab and Rituximab MAPPs prediction. **(A)** Infliximab MAPPs peptides were collected from Hamze et al. (15) and compared to in-house MAPPs profiles. Note that the collected dataset only contained peptides mapped to the variable regions of Infliximab light and heavy chains. NNAlign_MAC prediction promiscuity profiles and SCC correlation against the two datasets are shown in matching colors (red). **(B)** Scatter plot of the two MAPPs profiles [In-house vs. Hamze et al. (15)] for the heavy (red) and light chains (blue) of Infliximab protein-drug. **(C)** NNAlign_MAC correlation to the combined dataset [In-house + Hamze et al. (15)]. **(D)** Scatter plot of the NNAlign_MAC prediction vs the combined Infliximab MAPPs profile. **(E)** NNAlign_MAC correlation to Rituximab MAPPs data collected from Hamze et al. (15). **(F)** Scatter plot of the NNAlign_MAC prediction vs Rituximab MAPPs profile from the same publication.

Several protein regions, both in the light and heavy chain were predicted to have MHC-II ligands by NNAlign_MAC even though no peptides were identified in the MAPPs assays. We hypothesized that this was due to the sensitivity limitations of the MAPPs assay. To inspect this conjecture, additional Infliximab MAPPs data, from 21 and 16 donors covering the variable heavy and light chain regions of Infliximab, respectively, were collected from a previous publication (15). First, we evaluated the correlation of this new MAPPs dataset to the in-house Infliximab dataset (including only the variable region of the antibody) and observed in both cases a high (though lower for the light chain compared to the heavy chain) correlation between the two datasets (SCC = 0.662 and SCC = 0.842 for the light and heavy chain respectively) (Figures 4A,B). Given that no HLA-allele information was available to us for the donors used in this study, we evaluated the ability of NNAlign_MAC to predict the observed MAPPs using the alleles included in our in-house cohort, which have been selected covering the most frequent alleles in the world population (Supplementary Table 2) including only the variable regions of the protein (Figure 4A). Next, we combined the two Infliximab MAPPs datasets and analyzed the correlation of the NNAlign_MAC predictions to this new extended infliximab MAPPs dataset (Figures 4C,D). We found a substantial (*p*-value = 0.0003, bootstrap) increase in predictive performance with the SCC increased from 0.266 to 0.53 for the light chain, while the performance for the heavy chain was conserved (SCC changed from 0.952 to 0.924, *p*-value = 0.883) (Figure 4C) (similar results were obtained for MixMHC2pred). This observation suggests that the performance values of NNAlign_MAC reported in Figure 3A are lower bounds and that—at least some of—the additional peaks predicted by NNAlign_MAC represent regions with antigen presentation potential missed by the individual MAPPs assays.

As an additional proof of concept, we analyzed the correlation of NNAlign_MAC predictions to rituximab, an additional protein drug with MAPPs data collected from the above-mentioned publication (Figures 4E,F). The average SCC correlation considering heavy and light chains of both protein-drugs to the Hamze et al. (15) MAPPs data was 0.652 for NNAlign_MAC, showing that the proposed method was able to predict most of the MAPPs regions.

### NNAlign_MAC Is Able to Predict Infliximab-CD4 T Cell Epitopes

**Figure 5:**
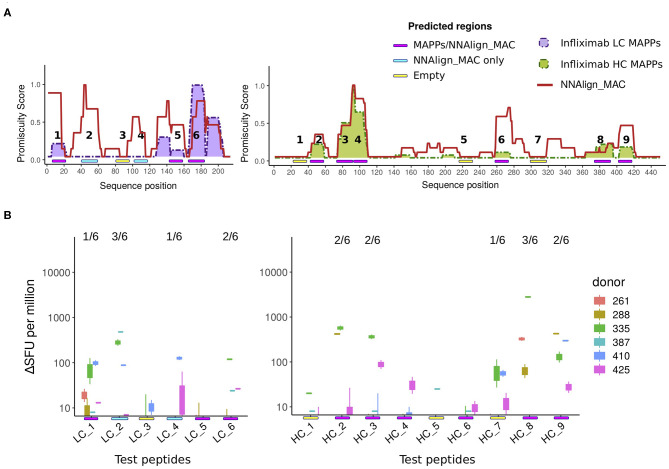
T cell evaluation of MAPPs and NNAlign_MAC identified hot-spot regions. **(A)** Schematic with of the location of 6 and 9 ELISpot tested peptides for the light and heavy chain of Infliximab, respectively. The peptides were identified with a color code covering three regions, (1) regions predicted by NNAlign_MAC where MAPPs peptides were found (Magenta: LC_1, LC_5, LC_6, HC_2, HC_3, HC_4, HC_6, HC_8, HC_9); (2) regions predicted by NNAlign_MAC, with no MAPPs peptides (Cyan: LC_2, LC_4); and regions were the methods identified none or very few ligands (Yellow: LC_3, HC_1, HC_5, HC_7). **(B)** IFN-γ ELISpot test for Infliximab peptides selected in **(A)**. Each boxplot was constructed from the two-individual donor-response measurement replicas to each peptide assessed. Units in IFN-γ production are expressed as counts ΔSFU per million (subtracting the average background for each donor assessment). The fraction number over each peptide line corresponds to the number of donors with a significant ELISpot response (4 times over the average background for the two independent measurements).

Next, we investigated if the peak regions predicted by NNAlign_MAC correlated with the location of CD4 T cell epitopes. For that purpose, 6 and 9 peptides respectively from Infliximab light and heavy chains were designed and assessed using ELISpot assays for CD4 T cell activation (for details refer to section Methods). These infliximab peptides were selected from three categories: MAPPs regions—covering regions predicted by NNAlign_MAC where MAPPs peptides were found (Figure 5A, Magenta: LC_1, LC_5, LC_6, HC_2, HC_3, HC_4, HC_6, HC_8, HC_9); NNAlign_MAC regions—regions predicted by NNAlign_MAC, with no MAPPs peptides (Figure 5A, Cyan: LC_2, LC_4); and regions were the methods identified none or very few ligands (Figure 5A, Yellow: LC_3, HC_1, HC_5, HC_7). In these assays, 67% (6/9) of the peptides spanning MAPPs positive regions were positive in at least one donor (all except for LC_5, HC_4 and HC_6, Figure 5B). Similar results were observed for the NNAlign_MAC region peptides (Figure 5B). Here, both peptides (LC_2, LC_4) were positive for at least one donor (Figure 5B). Finally, one of the peptides, HC_7, selected from the “empty” region (yellow) was found to give a marginal response in one of the assessed donors (Figures 5A,B). These findings thus demonstrate a very high correspondence between the NNAlign_MAC predictions, the location of observed T cell epitopes, and further suggest that MAPPs potentially can miss relevant regions leading to immunogenicity.

**Figure 6:**
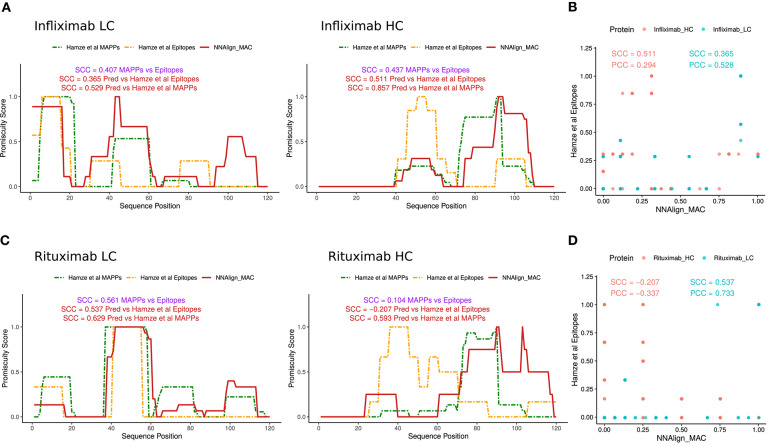
NNAlign_MAC is able to predict Infliximab-and Rituximab CD4 T cell epitopes. CD4 T cell epitope sequences identified by Hamze et al. (15) mapped to **(A)** Infliximab and **(C)** Rituximab variable regions of the light chain and heavy chain (orange dotted lines). NNAlign_MAC predicted profiles (Materials and Methods, profile generation) and SCC correlation to MAPPs are displayed in red. Scatter plot of the NNAlign_MAC prediction profiles vs MAPPs for **(B)** Infliximab and **(D)** Rituximab T cell responses from Hamze et al. (15).

As a final validation of the predictive power of the proposed prediction method, a set of CD4 T cell epitopes for Infliximab and Rituximab antibodies were collected from Hamze et al. (15). In this study, all 15mers spanning the light and heavy chain of the protein-drugs with an overlap of 10 amino acids were assessed in 15 healthy, 6 infliximab-treated donor, and 1 rituximab-treated donor, for T cell activation. Epitope profiles were constructed similarly to how MS profiles were built earlier by stacking the epitopes data over the light and the heavy chains of the protein-drugs and counting how many peptides overlap per each amino acid position. Next, as no complete allele information was provided for the tested donors, NNAlign_MAC predictions were made for the alleles present in our in-house MAPPs dataset, and promiscuity profiles were built for the light and heavy chain variable regions as described earlier (Figure 6). Notably, NNAlign_MAC was able to predict most of the T cell immunogenic regions for Infliximab (Figures 6A,B) in a comparable fashion to experimental MAPPs. Analyzing the hotspots regions in 30–45 from the light chain, or 90–105 from the heavy chain, we observe that NNAlign_MAC was able to predict those regions while the MAPPs experiment missed it (Figure 6A). In the Rituximab example, while both MAPPs (SCC = 0.561) and NNAlign_MA correlations (SCC = 0.537) show high and comparable performance for the light chain (*p*-value = 0.81, bootstrap), both approaches demonstrated very limited predictive power over Rituximab heavy chain epitopes (Figures 6C,D).

## Discussion

Here, we have constructed a predictor, NNAlign_MAC, for MHC class II antigen presentation trained on in-house MAPPs and data from the IEDB based on the previously developed NNAlign_MA machine learning framework (22, 23) integrating context information and HLA binding promiscuity scores. The predictor was demonstrated to vastly improve in performance over NetMHCIIpan for the prediction of MHC antigen presentation hotspots in protein drugs. Moreover, our findings strongly suggest that the use of such prediction methods could effectively serve as a complement to MAPPs assays to improve the sensitivity for identification of hotspot regions enriched in MHC ligands and T cell epitopes.

One of the strengths of the NNAlign_MAC algorithm lies in its ability to leverage information between multiple MAPPs datasets reducing noise and boosting performance in particular for alleles characterized by limited data (as exemplified by the clear motifs identified in the MAPPs data for the weakly expressed HLA-DR3, 4, 5 alleles). This combined with its pan-specific power (8) makes NNAlign_MAC less sensitive to the critical limiting issues often associated with MAPPs assays including the requirement of a massive amount of biological material, the need for experimental replicates and repeated assaying over HLA diverse cohorts, and the non-trivial task of interpreting/mapping the raw MS spectral data to genomic templates.

We have here demonstrated the power of NNAlign_MAC for two protein drugs infliximab and rituximab only. Further studies covering a broader set of proteins are needed to fully assess the gain in performance of prediction models trained on MS data for prediction of antigen presentation hotspots and T cell epitopes. Likewise, further studies are needed to assess if the complementary power observed in this study of *in-silico* predictions over MAPPs for hotspots identification remains valid when tested on a broader set of protein drugs. Moreover, additional methods for MHC-II antigen presentation prediction trained on MS data have recently been proposed (24–26). We showed an improved performance of NNAlign_MAC in predicting Infliximab MAPPS data compared to both NetMHCIIpan (5) and MixMHC2pred (25). Other methods have been recently published integrating MS MHC ligand data in the training. As for the method developed by Chen et al., MARIA (26), the comparison does not seem adequate in this scenario as its predictive power depends on the availability of protein expression levels, which makes limited sense in the context of protein-drugs. Another method, NeonMHC2 (24), only allows for to run max 20 predictions per day, making it impractical to include in a benchmark. Further evaluations remain to be conducted to benchmark the predictive power of these novel tools for the prediction of protein-drug MHC antigen presentation and immunogenicity. In conclusion, this work demonstrates that MS data can be used to train improved predictors for MHC class II antigen presentation, and showcase how such predictors can be used to effectively assess protein-drugs for the presence of MHC II hotspot and T cell epitope regions complementing the use of the conventional cost-intensive MAPPs assays.

## Data Availability Statement

The datasets generated for this study are available on ProteomeXchange Consortium via the PRIDE partner repository (http://www.ebi.ac.uk/pride) with the dataset identifier PXD018303.

## Ethics Statement

Donors IMXP00288 and IMXP00425 correspond to the protocol with national number B707201627607, IMXP00387 and IMXP00425 correspond to the protocol with national number B707201629385 and IMXP00335, IMXP00261 and IMXP00283 correspond to the protocol with national number NL5791207516. The full name and affiliations of the ethical committee for protocol NL5791207516 is METC Isala, Gebouw Mondriaan, Kamer 0.47, Postbus 10400, 8000 GK Zwolle, Netherlands. The full name and affiliations of the ethical committee for the two other protocols is Comité d'Ethique Hospitalo-Facultaire Universitaire de Liège (707), C.H.U. Sart Tilman, Domaine Universitaire du Sart Tilman, B35, 4000 Liège 1, Belgium.

## Author Contributions

CB, BR, and MN conducted research and developed the computational pipeline. CB, CA, LJ, SP, EP, and MN analyzed the data. AJ and CA performed sample processing. MW, SC-M, and J-PG generated the MAPPs data. CA and JS performed T cell assays. CB, SP, EP, and MN designed the study. CA, MW, SP, and EP provided methodological details and reviewed the manuscript. CB and MN wrote the paper.

## Conflict of Interest

MW, AJ, SC-M, J-PG, and EP are currently employees of Caprion Biosciences. CA, JS, and SP are employees of ImmunXperts. The remaining authors declare that the research was conducted in the absence of any commercial or financial relationships that could be construed as a potential conflict of interest.
