# Supplementary material for: Immunopeptidomic Data Integration to Artificial Neural Networks Enhances Protein-Drug Immunogenicity Prediction
Source: Front Immunol. 2020 Jun 23;11:1304. doi: 10.3389/fimmu.2020.01304 (PMC7325480; doi:10.3389/fimmu.2020.01304)
Supplement: Supplementary file 2 [file Data_Sheet_1.docx]

Supplementary Material

# Supplementary Figures and Tables

## Supplementary Tables

**Supplementary Table 1. HLA-DR Typing of healthy donors utilized in the in-house Infliximab MAPPs assay and IFN-γ-ELISpot T cell tests.**

**Supplementary Table 2. Peptides assessed for CD4 T cell responses.** Peptide sequences tested for IFN-γ-ELISpot T cell responses in Infliximab heavy and light chains.

**Supplementary Table 3. Cross-validation performances for NNAlign_MA models trained with and without PCI, and for NetMHCIIpan.** Performance values are presented in terms of AUC 0.1. For further details on cross-validation assessments for each method, refer to the Materials and Methods section.

**Supplementary Table 4. Evaluation of the CD4-T cell response by IFN-γ-ELISpot.** Analysis and statistics per peptide and donor.

## Supplementary Figures

**
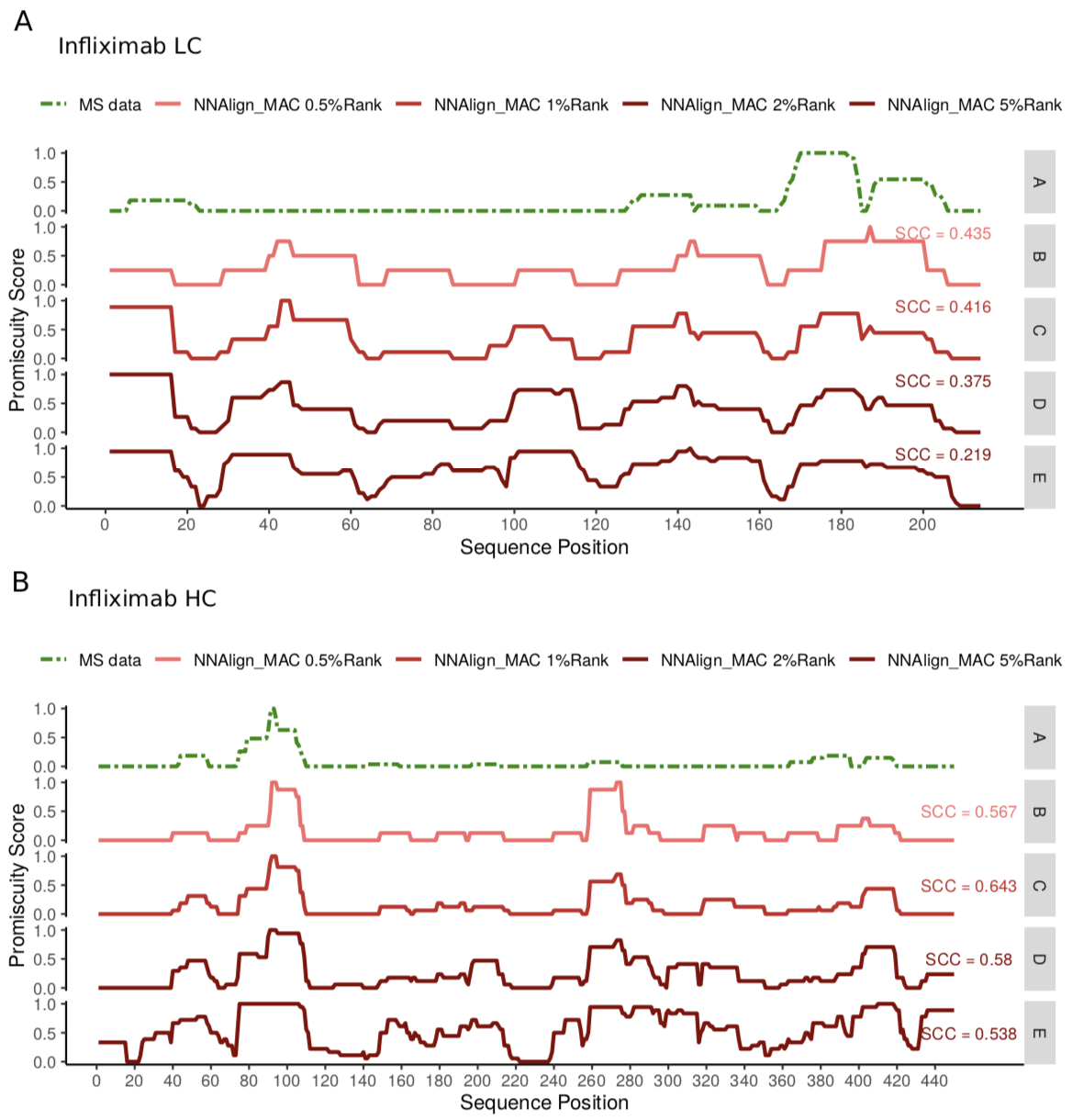
**

**Supplementary Figure 1. Comparison of NNAlign_MAC prediction profile schemes.** Promiscuity profile plots for different % Rank thresholds for **A.** light and **B.** heavy chains of Infliximab protein drug, applying the NNAlign_MAC prediction method. A Spearman correlation coefficient (SCC) is shown for each %Rank for both chains. Rank 1% showed the best SCC correlation to in-house Infliximab MAPPs and was therefore selected.

**
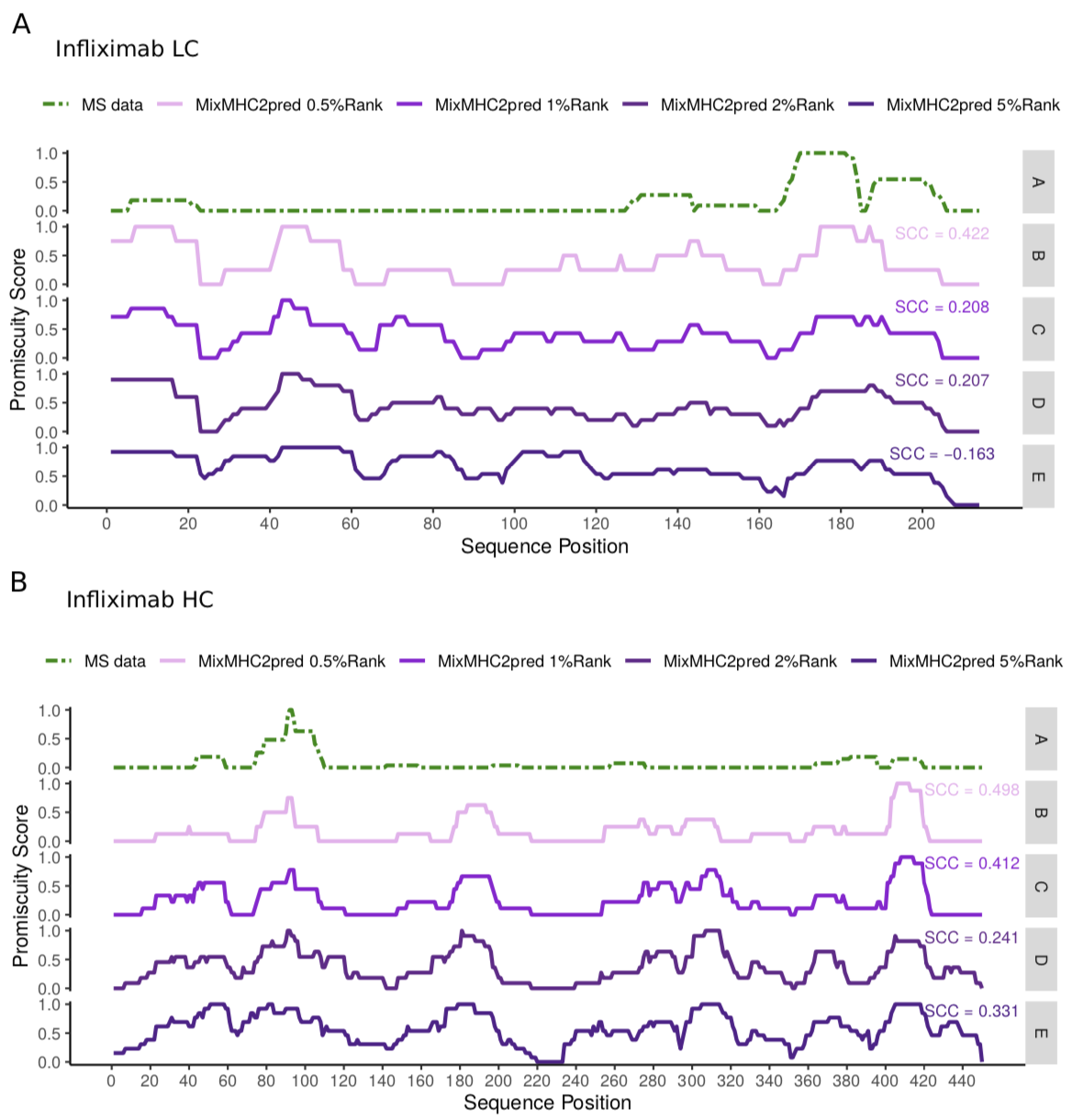
**

**Supplementary Figure 2. Comparison of MixMHC2pred prediction profile schemes.** Promiscuity profile plots for different % Rank thresholds for **A.** light and **B.** heavy chains of Infliximab protein drug, applying the MixMHC2pred prediction method. A Spearman correlation coefficient (SCC) is shown for each %Rank for both chains. Rank 0.5% showed the best SCC correlation to in-house Infliximab MAPPs and was therefore selected.

**
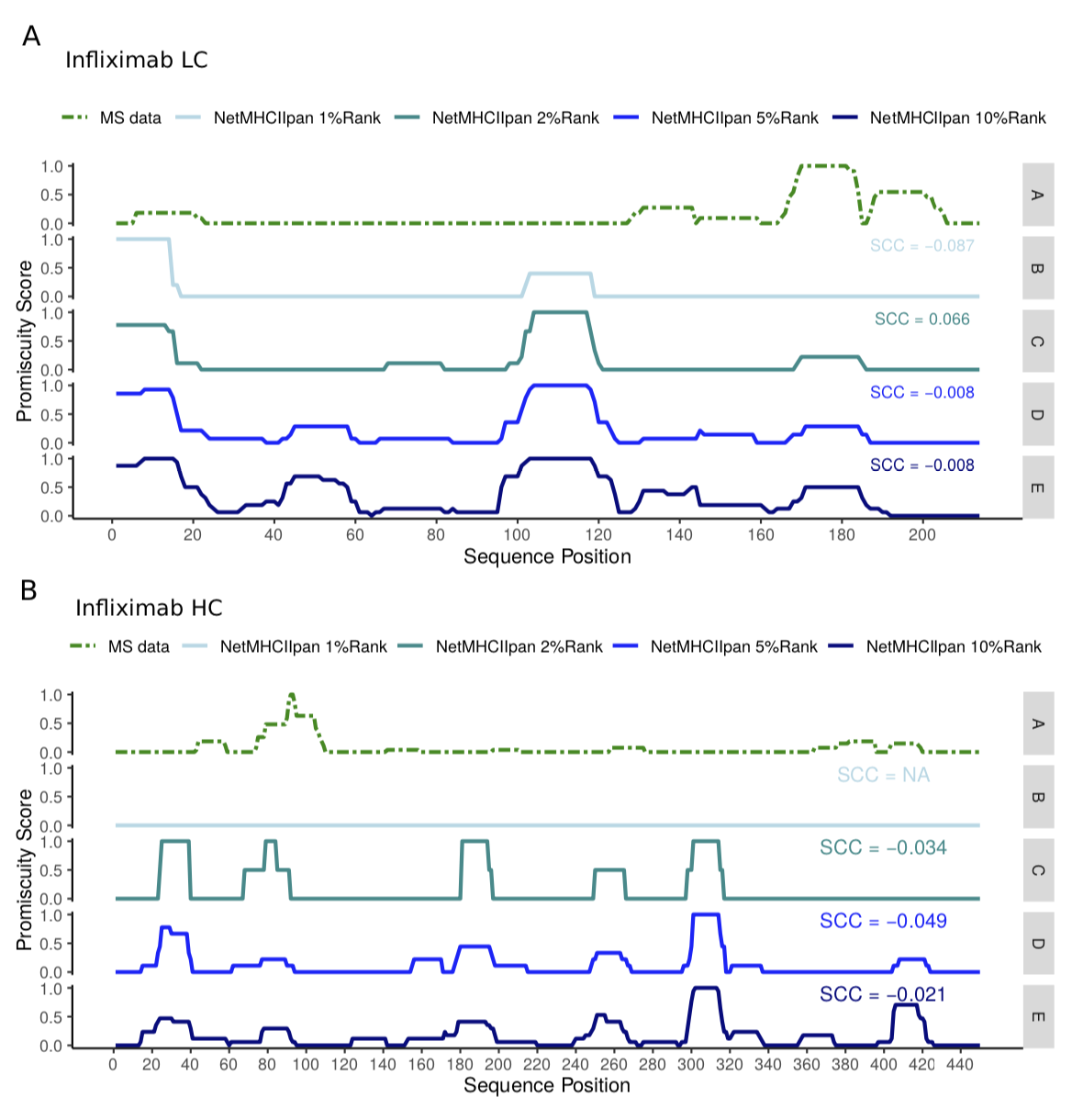
 Supplementary Figure 3. Comparison of NetMHCIIpan prediction profile schemes.** Promiscuity profile plots for different % Rank thresholds for **A.** light and **B.** heavy chains of Infliximab applying NetMHCIIpan prediction method. A Spearman correlation coefficient (SCC) is shown for each %Rank for both chains. To benchmark NetMHCIIpan a %Rank of 2 was selected. NNAlign_MAC and MixMHC2pred methods were evaluated at different and lower %Rank values. The prediction scores vary significantly because the models are trained on different data types (NetMHCIIpred on BA data, MixMHC2pred based on MS data, and NNAlign_MAC is both data types). Therefore, it is expected that each method will peak at different %Rank thresholds. To fairly compare these methods, different rank scores were assessed for NetMHCIIpan 3.2 including the percentual rank thresholds of 1, 2 (strong binder), 5 and 10 (weak binder).

**
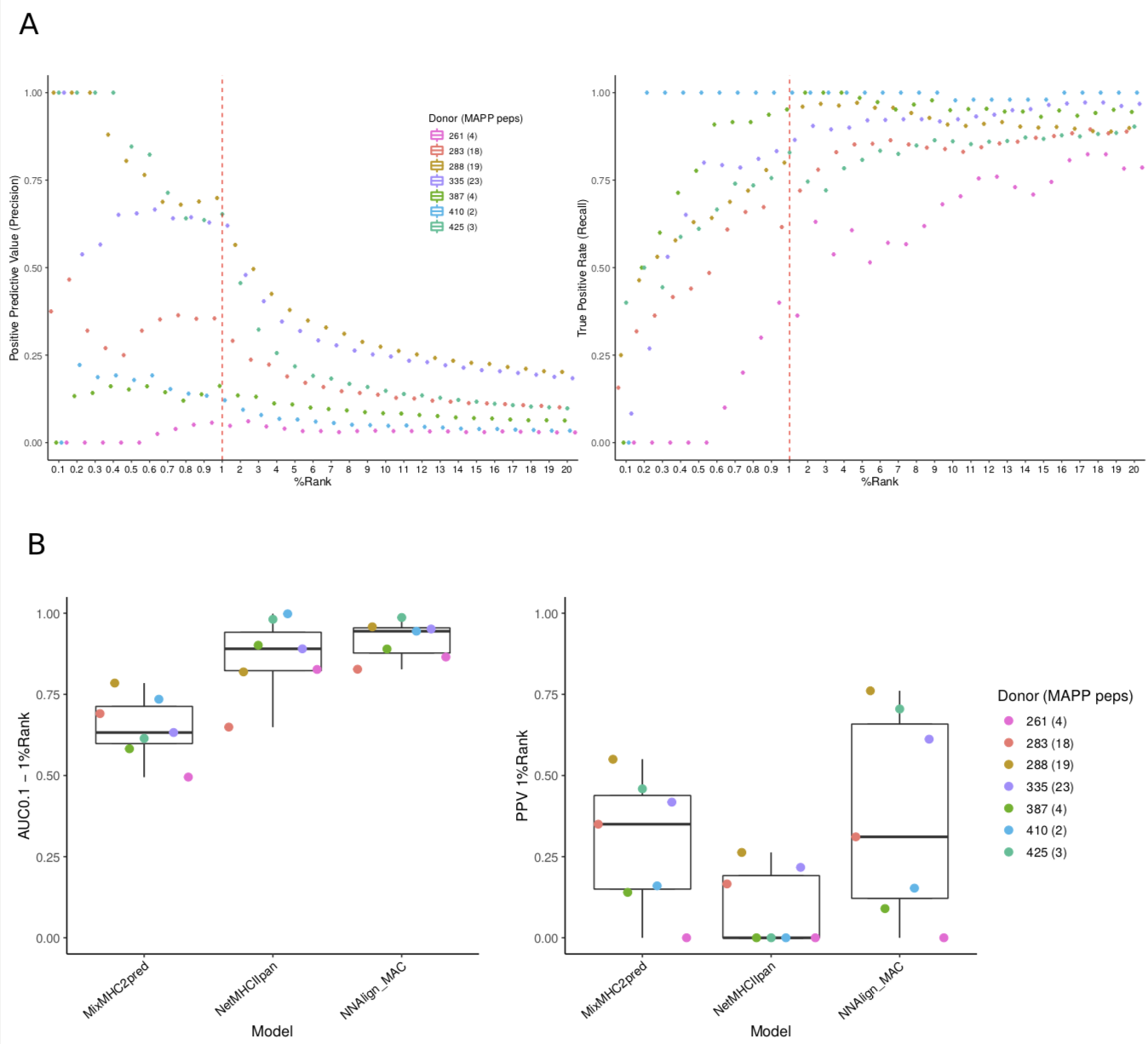
**

**Supplementary Figure 4. A.** NNAlign_MAC Precision and recall curves for different % Rank thresholds and different donors. The count of positive MAPPs peptides is displayed in parentheses for each of the MAPPs donors. Precision and recall curves were constructed using a relaxed-core approach described in detail in Materials and Methods. **B.** AUC0.1 and PPV performance measures for MixMHC2pred, NetMHCIIpan and MixMHC2pred for the prediction of Infliximab in-house MAPPs peptides per donor. Values in parenthesis indicate the number of Infliximab MAPPs peptides found for each donor.
